# A phase I-II trial of fludarabine, bendamustine and rituximab (FBR) in previously treated patients with CLL

**DOI:** 10.18632/oncotarget.12054

**Published:** 2016-09-15

**Authors:** Nitin Jain, Kumudha Balakrishnan, Alessandra Ferrajoli, Susan M. O’Brien, Jan A. Burger, Tapan M. Kadia, Jorge E. Cortes, Mary L. Ayres, Francesco Paolo Tambaro, Michael J. Keating, Varsha Gandhi, William G. Wierda

**Affiliations:** ^1^ Department of Leukemia, The University of Texas MD Anderson Cancer Center, Houston, TX, USA; ^2^ Department of Experimental Therapeutics, The University of Texas MD Anderson Cancer Center, Houston, TX, USA; ^3^ The UCI Chao Family Comprehensive Cancer Center, Orange, CA, USA; ^4^ S.S.D. TMO - AORN Santobono-Pausilipon, Napoli, Italy

**Keywords:** chemoimmunotherapy, fludarabine, bendamustine, rituximab, CLL

## Abstract

Chemoimmunotherapy regimens have been the standard first-line therapy for patients with chronic lymphocytic leukemia (CLL). For young, fit patients the standard of care is combination of fludarabine, cyclophosphamide, and rituximab (FCR). Based on the preclinical work demonstrating that bendamustine combined with fludarabine resulted in increased DNA damage, we designed a phase I-II clinical trial with fludarabine, bendamustine, and rituximab (FBR) for patients with relapsed/refractory CLL. Treatment consisted of fludarabine 20 mg/m^2^ daily x 3 days and rituximab 375-500 mg/m^2^ x 1 day. Phase I included bendamustine at increasing doses of 20, 30, 40, or 50 mg/m^2^ daily x 3 days; phase II was with FR, and B at the selected dose. DNA damage response (H2AX phosphorylation) was evaluated in a subset of patients. Fifty-one patients were enrolled. The median age was 62 years; median number of prior therapies was 2; 40% had del(11q); and 41 patients had received prior FCR-based therapies. Hematologic toxicity was more common in =40 mg/m^2^ dose cohorts. Maximum tolerated dose (MTD) was not identified. Bendamustine-elicited H2AX phosphorylation was not dose-dependent, but markedly increased after fludarabine. We identified bendamustine 30 mg/m^2^ as the safe dose for phase II. The overall response rate (ORR) was 67% with 36% complete response (CR) / CR with incomplete count recovery (CRi). Younger patients (<65 years) had significantly higher ORR (81% *vs*. 50%; *p*=0.038). The median progression-free survival was 19 months, and the median overall survival was 52.5 months. FBR is an effective and tolerable CIT regimen for patients with relapsed CLL.

## INTRODUCTION

Chemoimmunotherapy (CIT) regimens such as a combination of fludarabine, cyclophosphamide, rituximab (FCR) have been the standard first-line therapy for younger fit patients with chronic lymphocytic leukemia (CLL). [1] Our group previously reported an overall response rate (ORR) of 95% with a complete remission (CR) rate of 72% in patients treated with the FCR regimen in the first-line setting. [2, 3] The German CLL Study Group (GCLLSG) CLL8 trial, a randomized study of FCR vs. FC (fludarabine/cyclophosphamide) confirmed the superiority of FCR in the first-line setting with a higher CR rate, and longer progression-free survival (PFS), and overall survival (OS). [4] In patients with relapsed CLL, FCR was shown to be an effective salvage regimen. [5] The REACH trial, showed superiority of FCR over FC in patients with relapsed/refractory (R/R) CLL. [6] These trials help establish the role of CIT in the management of patients with CLL.

Bendamustine, an alkylating agent, has a complex structure with 3 functional groups: an alkylating group, a benzimidazole ring, and a butyric acid side chain. [7] It has alkylating-agent activity, capable of inducing inter-strand and intra-strand DNA cross-links. [8, 9] The GCLLSG reported on the outcomes of bendamustine combined with rituximab (BR) in patients with R/R CLL. [10] Bendamustine was administered at a dose of 70 mg/m^2^ on days 1 and 2 combined with rituximab 375 mg/m^2^ x1 during the first course and 500 mg/m^2^ x1 during courses 2-6. The ORR noted was 59% with a CR rate of 9%. The median PFS was 15.2 months. Grade (G) 3 or G4 thrombocytopenia, neutropenia, and anemia were documented in 28%, 23%, and 17% of patients, respectively. Bendamustine is currently approved for the management of patients with CLL.

Work done by our group [11–13] demonstrated that DNA excision repair is inhibited by fludarabine, providing a rationale for combining alkylating agents such as cyclophosphamide with a purine analog such as fludarabine. [14] The addition of a CD20 monoclonal antibody (mAb) further improved the efficacy of the combination of purine analog and alkylating agents. [4, 6] Our recent investigations in primary CLL lymphocytes demonstrated that, similar to cyclophosphamide, bendamustine in combination with fludarabine resulted in an increased DNA damage response and augmented biological consequences. [15] These were maintained even when malignant lymphocytes were interacting with the protective stromal microenvironment. We thus hypothesized that the combination of fludarabine, bendamustine, and rituximab (FBR regimen) would be synergistic. [16] In 2009, prior to the introduction of oral targeted therapies in CLL, we initiated a phase I-II clinical trial with FBR in patients with relapsed and/or refractory (R/R) CLL. The primary objective of the phase I part was to identify the maximum tolerated dose (MTD) of bendamustine combined with fludarabine and rituximab in R/R CLL. The primary objective of the phase II part was to assess the efficacy of the regimen. Secondary objectives included pharmacodynamic endpoints to determine bendamustine-induced DNA damage response in CLL lymphocytes and test the hypothesis that fludarabine triphosphate will enhance this response by inhibiting DNA repair. We report here final results of the FBR regimen in patients with R/R CLL.

## RESULTS

### Patient characteristics

A total of 51 patients with R/R CLL were enrolled on this trial between April 2010 and December 2013. The median age of the patients was 62 years (range, 46-82 years). Twenty-four (47%) patients had Rai stage III-IV disease. Baseline characteristics are summarized in Table 1. The median number of prior therapies was 2 (range, 1-6). Details of prior therapies are in Supplementary Table 1. Of the 51 patients, 41 received prior FCR-based therapies. All patients had prior CD20 mAb therapy. One patient had a prior allogeneic stem cell transplant (allo-SCT). A total of 40% of the patients had del(11q). The median number of treatment courses FBR administered was 3 (range, 1-6). The median follow-up is 31 months (range, 7.9-61.3 months).

**Table 1:** Patient Characteristics

Characteristic (*N*= 51)	Value
Age (years), median (range) Age ≥65 years, *n* (%)	62 (46-82) 22 (43)
Median number of prior therapies, (range)	2 (1-6)
Rai Stage III-IV, *n* (%)	24 (47)
β-2M (mg/l) (*n* = 50) median (range) β-2M ≥ 4 mg/l, n (%)	4 (1.8-10.4) 25 (50)
ALC (K/μl), median (range)	12.5 (0.1-220)
Hemoglobin (gm/dl), median (range)	12.9 (8.4-14.7)
Platelet count (K/μl), median (range)	103 (15-414)
*IGHV* Unmutated (*n* = 42), *n* (%)	33 (79)
CD38 Positive (≥30%), *n* (%)	32 (63)
FISH (*n* = 45), *n* (%) del(17p) del(11q) Trisomy 12 No FISH abnormality detected del(13q)	5 (11) 18 (40) 9 (20) 8 (18) 5 (11)

ALC: Absolute Lymphocyte Count; IGHV: Immunoglobulin heavy chain variable region; FISH: Fluorescence *in situ* hybridization

### Dose-escalation phase

A total of 21 patients were enrolled in phase I [bendamustine dose level 20 mg/m^2^ (*n* = 6); 30 mg/m^2^ (*n* = 3); 40 mg/m^2^ (*n* = 6); 50mg/m^2^ (*n* = 6)]. Course 1 toxicities were predominantly G1-2 and most common were nausea, fatigue, and hyperglycemia. One of 6 patients experienced DLT (G3 nausea/vomiting/dehydration) in the 20 mg/m^2^ cohort; 0 of 3 pts experienced DLT in the 30 mg/m^2^ cohort; 1 of 6 patients experienced DLT (G4 sepsis) in the 40 mg/m^2^ cohort; and 1 of 6 patients experienced DLT (G4 sepsis, renal failure) in the 50 mg/m^2^ cohort. Hematologic toxicity was more common in the 40 and 50 mg/m^2^ cohort with G4 thrombocytopenia noted in 17% and 33% of patients, respectively (Supplementary Table 2). There were no treatment-related deaths. No MTD was identified in the phase I. Given more frequent adverse events at the higher dose levels, particularly during later courses, we identified bendamustine 30 mg/m^2^ as the safe and active dose level to expand in phase II. An additional 30 patients were enrolled on the 30 mg/m^2^ dose cohort.

### Clinical responses

**Figure 1:**
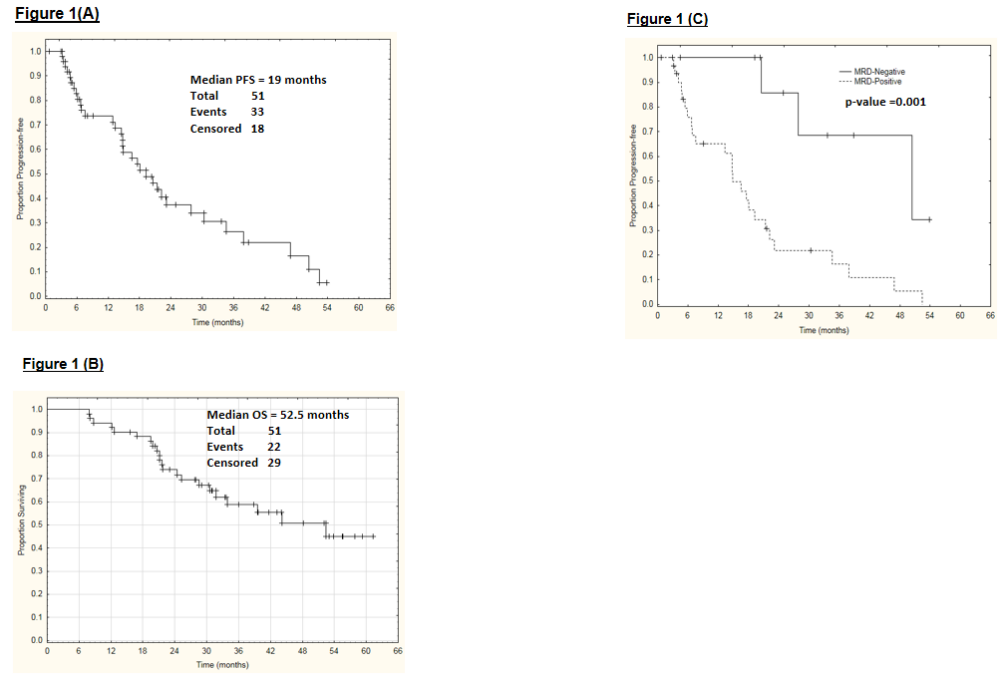
**A**. Progression-free survival of all patients, **B**. Overall survival of all patients, and **C**. Progression-free survival based on minimal-residual disease (MRD) remission status

A total of 49 patients were evaluable for response (2 patients were not evaluable due to loss to follow-up). In an intent-to-treat analysis (*n* = 51), the ORR was 67% with 36% CR/CRi (20% CR, 16% CRi) and 31% PR (Table 2). At the MTD (30 mg/m^2^ dose; *n* = 33), the ORR was 68% with 27% CR/CRi and 39% PR. A total of 10 patients (7 CR; 2 CRi; 1PR) achieved MRD-negative remission by multicolor flow-cytometry (at least 10^−4^ sensitivity) in the bone marrow. Of the 10 patients who achieved CR, 7 (70%) achieved MRD-negative remission. Clinical responses by pretreatment characteristics are reported in Table 3. Younger patients (age < 65 years) had significantly higher ORR (81% *vs*. 50%; *p* = 0.038). Patients with low β-2M ( < 4 mg/l) had higher ORR (80% vs. 52%, *p* = 0.07). Notably, 5 of the 9 patients with trisomy 12 achieved MRD-negative CR. Clinical responses by bendamustine dose level are summarized in Table 3. The median PFS was 19 months (Figure 1A), and the median OS was 52.5 months (Figure 1B). Patients achieving MRD-negative remission had significantly improved PFS (*p* = 0.001) (Figure 1C). Three patients (6%) developed therapy-related myelodysplastic syndrome (MDS) or acute myeloid leukemia (AML).

**Table 2:** Responses

Response Category (*N*= 51)	No. (%) Patients	MRD Negative
Overall Response (ORR)	34 (67)	10/34
Complete Remission (CR)	10 (20)	7/10
CRi	8 (16)	2/8
Partial Remission (PR)	16 (31)	1/16
Fail	15 (29)	
Not Evaluable	2 (4)	

MRD: Minimal Residual Disease

**Table 3:** Responses by Pretreatment Characteristics and Bendamustine Dose

Pretreatment Characteristic	N	%CR/CRi	%PR	%ORR
Age ≥65 yrs <65 yrs	22 29	27 41	23 38	50 81
Rai Stage III-IV 0-II	24 27	42 30	25 37	67 67
Number of Prior Therapies ≥3 ≤2	22 29	32 38	27 34	59 72
Fludarabine-refractory Fludarabine-sensitive	4 47	0 38	25 32	25 70
β-2M ≥4 mg/l <4 mg/l	25 25	28 44	24 36	52 80
*IGHV* Unmutated Mutated	33 9	33 67	33 11	66 78
CD38 ≥30% <30%	32 19	34 37	38 21	72 58
FISH del(17p) del(11q) Trisomy 12 No FISH abnormality detected del(13q)	5 18 9 8 5	20 28 67 37 20	80 44 22 0 20	100 72 89 37 40
Bendamustine Dose 20 mg/m^2^ 30 mg/m^2^ 40 mg/m^2^ 50 mg/m^2^	6 33 6 6	50 27 66 33	33 39 17 0	83 68 83 33

### Toxicities

Hematologic toxicity was common. G3-4 neutropenia was seen in 76% patients (G3 16%, G4 60%), and G3-4 thrombocytopenia was seen in 49% patients (G3 20%, G4 29%). G3-4 anemia was seen in 22% of the patients. A total of 48% of patients received G-CSF, either as prophylaxis or for management of prolonged neutropenia/neutropenic fever. G≥3 infection was noted in 33% of patients. A total of 26% of patients needed dose reduction, most commonly for prolonged myelosuppression.

### Induction of DNA damage response by bendamustine-(H2AX phosphorylation)

Circulating CLL cells from 15 patients at different doses of bendamustine 20 mg/m^2^ (*n* = 3); 40 mg/m^2^ (*n* = 3); 50 mg/m^2^ (*n* = 2) (phase 1) and 30 mg/m^2^ (*n* = 7, phase 2) were evaluated for pharmacodynamic endpoints. The DNA damage response was assessed by measuring H2AX phosphorylation at 2, 4, and 6 hours after start of bendamustine. Compared to base-line value, there was an increase in H2AX phosphorylation that peaked by 4 hrs after start of bendamustine. Considering the response in control or pretreated samples as 1-fold, the increase during combination ranged between 1-29 fold; (Supplemental Figure 1; *n* = 15).

**Figure 2:**
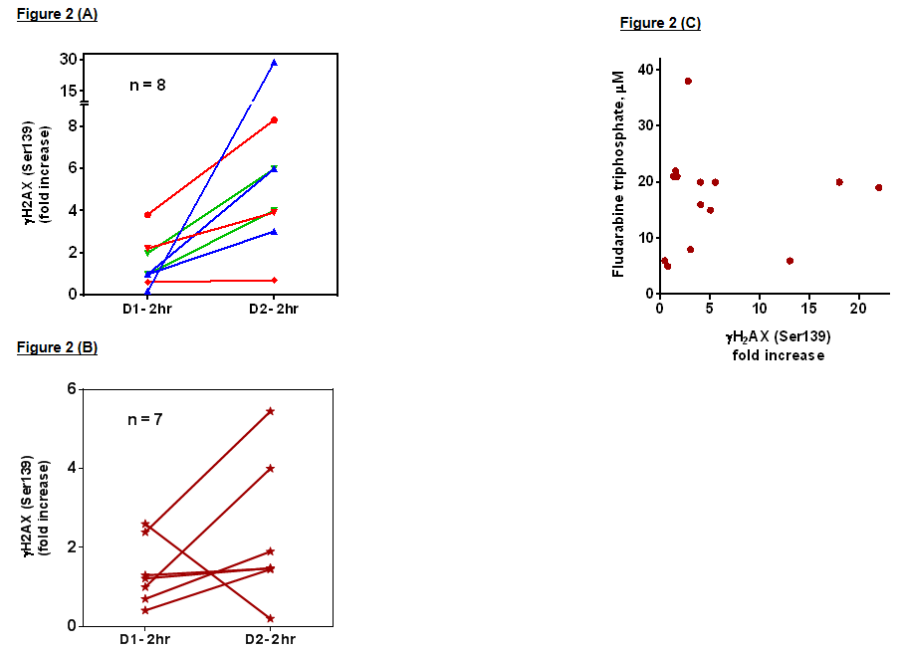
**A**. **DNA damage response measured by H2AX phosphorylation**: Blood samples were obtained pre- and 2 hrs post-therapy on day 1 (bendamustine alone) and day2 (bendamustine with fludarabine) and CLL lymphocytes were isolated by ficoll gradient method and fixed with ice-cold ethanol (70%) and 4% PFA/PBS. The H2AX phosphorylation (γH2AX) was determined by flow cytometry method (*n* = 15) as described in materials and methods. Pretreatment value was taken as one and fold-increase in H2AX signal was plotted. A. Patients on phase I study with 20 (red), 40 (blue), and 50 (green) mg/m^2^ bendamustine. B. Patients on phase II study with 30 mg/m^2^ of bendamustine. **B**. **Correlation between fludarabine triphosphate levels and DNA damage response**. The DNA damage response measured by H2AX phosphorylation in figure 4 was correlated with fludarabine triphosphate levels for each patient sample. Fludarabine triphosphate was measured by HPLC 4 hrs after start of fludarabine infusion. H2AX value was at 4 hrs after start of bendamustine on day 2. For H2AX, the values are fold increase with pretreatment phosphorylation taken as one.

Of note, bendamustine neither as a single agent nor in couplet with fludarabine exhibited dose-dependent increases in DNA-damage response (Figure 2A and 2B). However, addition of fludarabine to bendamustine illustrated an increase in H2AX phosphorylation in 11 of 15 samples (Figure 2A and 2B is the comparison between 2 hr post bendamustine with (day 2) and without fludarabine (day 1) at different dose levels of bendamustine).

The median intracellular fludarabine triphosphate level at the start of bendamustine infusion was 20 μM (range 5-40 μM; Figure 2C; *n* = 14). This was sufficient to increase H2AX phosphorylation response (Supplemental Figure 1; *n* = 15). Correlation analysis between H2AX phosphorylation and fludarabine triphosphate levels for each patient revealed a non-linear correlation between these two end-points (Figure 2C).

**Figure 3:**
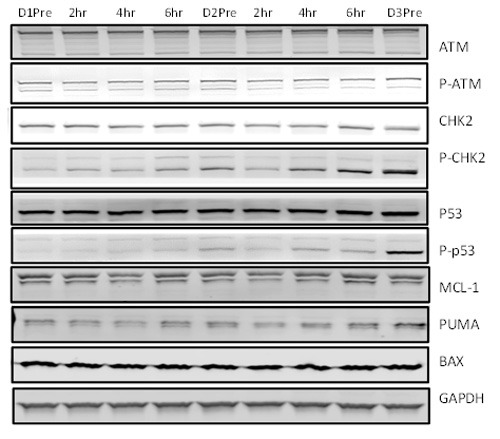
Stabilization of proteins ATM and p53 following DNA damage response: CLL lymphocytes obtained pre- and post-therapy were lysed and immunoblotting assay was performed for DNA damage proteins and anti-apoptotic proteins as described in materials and methods One representative patient data is provided.

Molecular markers of DNA damage response and cell death (ATM, CHK-2, p53, Mcl-1) were evaluated. Immunoblotting analysis demonstrated an increase in DNA damage response following fludarabine treatment that is evidenced by stabilization of response proteins ATM and p53 (Figure 3; *n* = 2). Puma was increased, but no change was observed with Mcl-1 and Bax.

## DISCUSSION

*In vitro* investigations revealed that fludarabine combined with bendamustine enhanced apoptosis in comparison to single-agent exposure. [15] We report here results of FBR CIT in patients with R/R CLL. The FBR regimen was tolerated up to the highest bendamustine dose evaluated during first course, with significant efficacy in previously treated patients with CLL.

This is the first study to identify the recommended phase II dose of bendamustine as 30 mg/m^2^ for 3 days combined with fludarabine 20 mg/m^2^ daily for 3 days and rituximab 375 mg/m^2^. This was based on first, the observed cumulative myelosuppression. Second, the overall response rates (including CR rate) were not dependent on bendamustine-dose (Table 3). Third, bendamustine-induced DNA damage response was dose-independent. (Figure 2A and 2B). We observed an ORR of 67% with a CR/CRi rate of 36% in a heavily pretreated patient population. Notably, 80% of the patients had prior FCR-based chemotherapy. These data compare favorably to ORR of 60-70% and CR/CRi rate of 25-30% noted with other CIT regimens in the relapsed setting (Supplementary Table 3). [5, 6, 10, 17–20] There was a relatively large proportion of patients (40%) with del(11q) in this trial. Overall, there was no statistical difference in the response rates achieved in various FISH subgroups. Notably, patients with trisomy 12 appeared to have the highest likelihood of achieved a MRD-negative CR (5 of the 9 patients achieving MRD-negative CR). Adding fludarabine to bendamustine resulted in increased DNA damage response in the majority of the patients tested (Figure 2). The FBR regimen has also been used as a non-myeloablative conditioning regimen for allo-SCT in patients with lymphoma and CLL. [21]

Toxicities with FBR were comparable to those seen with other CIT in the relapsed setting (Supplementary Table 3). G3-4 neutropenia was seen in 76% patients, and G3-4 thrombocytopenia was seen in 49% patients.

With the caveat of the challenges of inter-trial comparisons, the FBR regimen appeared to have higher CR/CRi rate than that seen with the BR regimen (Supplementary Table 3). In the GCLLSG BR trial, the CR/CRi rate was only 9% compared to 36% noted with the FBR regimen. [10] Notably, there were more patients with high-risk FISH and unmutated *IGHV* in the FBR group. Hematologic toxicities and infection risk appeared to be higher with the FBR regimen compared to those seen with the BR regimen. Only a randomized clinical trial can compare the role of FBR vs. BR or FCR in relapsed CLL. Such a trial however, is unlikely to be done in the current era of targeted therapy. Preclinical *in vitro* data in primary CLL cells suggest an increase in sensitivity of cells to bendamustine when fludarabine is combined [15].

CIT is the standard treatment approach for first-line treatment of patients with non-del(17p) CLL. Up until the approval of ibrutinib and idelalisib in patients with relapsed CLL, CIT was also the standard treatment for patients with relapsed CLL. These novel targeted therapies provide a safer, and likely more effective approach to treating patients with CLL as compared to CIT. This is currently being studied in several phase III trials. [1] The role of CIT in relapsed CLL has diminished, and it is likely CIT will be reserved for patients who have failed targeted therapies. We report that FBR is an effective and tolerable CIT regimen for patients with relapsed CLL. Current strategies are investigating CIT combinations with targeted B cell receptor signaling pathway inhibitors like ibrutinib and idelalisib; FBR appears to be a strong potential partner for combination study.

## MATERIALS AND METHODS

### Study group

Patients with R/R CLL requiring treatment per IWCLL 2008 criteria [22] were enrolled in this phase I-II trial. Patients must have had performance status (Eastern Cooperative Oncology Group) 0-2, and adequate liver and renal function [serum creatinine ≤2 mg/dL, alanine aminotransferase (ALT) ≤ 2.5 times upper limit of normal (ULN), total bilirubin ≤ 2.5 times ULN]. Patients with uncontrolled life-threatening infections were excluded. The MD Anderson Cancer Center Institutional Review Board approved this study; patients provided written informed consent per institutional guidelines, and this study was conducted in accordance with the Declaration of Helsinki. This trial was registered on Clinicaltrials.gov (#NCT01096992).

### Treatment

In phase I, the treatment consisted of fixed-dose fludarabine 20 mg/m^2^ daily x 3 days (days 2-4 during course 1, and days 1-3 during courses 2-6) and rituximab 375 mg/m^2^ x 1 day (day 4 during course 1) and 500 mg/m^2^ x 1 day (day 1 during each of courses 2-6), with bendamustine at increasing doses of 20 mg/m^2^ daily x 3 days, 30 mg/m^2^ daily x 3 days, 40 mg/m^2^ daily x 3 days, or 50 mg/m^2^ daily x 3 days. To investigate the benefit of adding fludarabine to bendamustine with respect to bendamustine-induced DNA damage, the pharmacological end points were investigated during course 1 (days 1-3), in which the treatment started with single agent bendamustine on day 1. On days 2 and 3 fludarabine was infused first and 2 hrs later bendamustine was administered. Unlike course 1, courses 2 - 6 consisted of the bendamustine and fludarabine couplet through days 1-3. Courses were repeated every 4 weeks or later if required for hematopoietic recovery; a maximum of 6 courses were given. Dose reduction was permitted for G3 or G4 neutropenia-associated infection or organ toxicity or if patients did not have adequate hematologic recovery by 42 days after the last course. Allopurinol was recommended for at least the first week of course 1 for tumor lysis prophylaxis. Valacyclovir (or acyclovir) was recommended for herpes virus prophylaxis, and trimethoprim/ sulfamethoxazole DS for PCP prophylaxis. G-CSF support was allowed but not mandated.

Patients were evaluated at enrollment with a baseline complete blood count, β2-microglobulin, bone marrow aspirate, and biopsy, including flow cytometry. Prognostic markers such as fluorescence *in situ* hybridization (FISH), ZAP-70 and CD38 expression, and *IGHV* mutation status were assessed at baseline. Complete blood count and biochemical assessment were performed before each course, and response assessment with physical examination, complete blood count, bone marrow aspirate and biopsy, and CT imaging was performed prior to course 4, and 3-months after the last course of FBR. MRD was evaluated by 4-color flow cytometry with sensitivity of 10^−4^. Response assessment was based on IWCLL 2008 criteria. [22] Toxicity was assessed according to Common Terminology Criteria for Adverse Events (CTCAE) v4.03.

### Collection and isolation of blood mononuclear cells

Whole blood was collected in heparinized tubes at indicated time points from Day 1 to Day 3. The lymphocytes were isolated by Ficoll-Hypaque gradient method (specific gravity, 1.086; Life Technologies, Grand Island, NY) as described previously. [23] CLL cells were washed twice with cold phosphate buffered saline (PBS) and a coulter channelyzer (Coulter Electronics, Hialeah, FL) was used to count cell number and mean cell volume.

### Measurement of H2AX phosphorylation

The lymphocytes obtained pre- and post-therapy were washed with PBS and fixed in 6 to 8 mL ice-cold ethanol (70%) and stored at −20°C until analysis for H2AX phosphorylation on the flow cytometry. The cells were then fixed with 4% fresh paraformaldehyde/PBS (pH 7.4) at room temperature (RT) for 10 min. After 2 washes with 1% BSA/PBS, the cells were blocked by gently shaking with PBS containing 5% GS (goat serum) /1% BSA at RT for 1 hr. This was followed by incubation with anti-phospho-Histone H2AX (Ser139) Antibody, clone JBW301, FITC conjugate (mouse monoclonal) (16-202A; Upstate, Billerica, MA) for 2 hours (gentle shake). The labeled cells were washed twice with cold PBS and resuspended in 1 mL of PBS containing the counterstain propidium iodide (15 μg/mL) and RNAase (Roche, South San Francisco, CA) (2.5 μg/mL) and incubated in the dark for 5 minutes at 37°C before analysis using FACScalibur (BD Biosciences, San Jose, CA). Data were expressed as fold increase of H2AX phosphorylation.

### Intracellular fludarabine triphosphate quantification

Nucleotides were extracted using 0.4 N perchloric acid and were separated on an analytical ion-exchange column (Partisil 10 SAX, 4.6 × 250 mm; Whatman, Maidstone, England) and quantified using external authentic standards. The intracellular concentrations of nucleotides were calculated using total cell count and the median cell volume [24].

### Immunoblot analysis

Cell pellets were lysed in RIPA lysis buffer and the protein content was determined using a DC protein assay kit (Bio-Rad Laboratories). The proteins were run on electrophoresis gels and transferred to nitrocellulose membranes (GE Osmonics Labstore) as described previously. [24] Membranes were blocked for 1 hour in blocking buffer, incubated with primary antibodies against total ATM (ab17995; Abcam, Cambridge, MA), phospho-ATM^Ser1981^ (EMD Millipore, Billerica, MA), total p53 (EMD Millipore, Billerica, MA), phospho-p53^Ser15^ (05-740; Cell Signaling Technology, Danvers, MA), CHK-2 (3440) and phospho-CHK-2^Thr68^ (2661; Cell Signaling Technology, Danvers, MA), MCL-1 (AHO0102; Life Technologies Corporation, Carlsbad, CA), PUMA (3043; ProSci Incorporated, Poway CA), BAX (sc-20067; Santa Cruz Inc, Dallas, TX) and GAPDH (5174; Cell Signaling Technology, Danvers, MA).

Following washing with PBST, membranes were incubated for 1 hour with infrared dye-labeled secondary antibodies (LI-COR Biosciences), scanned, and visualized using a LI-COR Odyssey infrared imager.

### Statistical analysis

A standard 3+3 design was used to evaluate for tolerability and toxicities of combined bendamustine (increasing doses), fludarabine, and rituximab. The dose-limiting toxicity (DLT) and MTD were evaluated based on the first course of the FBR regimen. DLT was defined as treatment-related, G≥3 non-hematologic toxicity. Hematologic toxicity G≥3 that lasted > 42 days was also considered a DLT. Four dose levels for bendamustine were investigated (20, 30, 40 and 50 mg/m^2^ daily for 3 days). MTD was defined as the highest dose level in which 6 patients have been treated with ≤ 1 patient experiencing DLT. In the phase II, the efficacy was assessed using the Simon's two stage MinMax design. The treatment were to be considered promising if the CR rate was 40% or higher, and be considered unworthy of further investigation if the CR rate was 25% or lower. Actuarial survival and PFS were estimated using the methods of Kaplan and Meier. Fisher exact test (2-tailed) was used to analyze differences in response outcomes by pretreatment characteristics. Linear regression analysis was performed using the GraphPad Prism 6 software (GraphPad Software, Inc. San Diego, CA).

### Research support

Supported in part by grant CA81534 from the National Cancer Institute, Department of Health and Human Services.

## SUPPLEMENTARY MATERIALS FIGURES AND TABLES


