# Supplementary material for: A phase I-II trial of fludarabine, bendamustine and rituximab (FBR) in previously treated patients with CLL
Source: Oncotarget. 2016 Sep 15;8(13):22104–12. doi: 10.18632/oncotarget.12054 (PMC5400650; doi:10.18632/oncotarget.12054)
Supplement: Supplementary file 1 [file oncotarget-08-22104-s001.pdf]

A phase I-II trial of fludarabine, bendamustine and rituximab (FBR) in previously treated patients with CLL

Supplementary Material

Supplementary Table 1. Prior Therapies Received

FC, **FCR**
Rituximab, **FCR**, Rituximab/steroids, OFAR, 8-chloroadenosine
**FCR**, CD40 mAb, **CFAR**, Lenalidomide/rituximab
**FCR** (15 patients)
Lenalidomide, **FCR**
**FCR**, Alemtuzumab
Fludarabine, **FCR**, Rituximab, Alemtuzumab
CHOP, ibritumomab tiuxetan, Rituximab/alemtuzumab, lenalidomide
**FCR**, OFAR, 8-chloroadenosine
**FCR/mitoxantrone**
Chlorambucil, Rituximab/steroids, **FCR**
**CFAR** (2 patients)
RCHOP, Rituximab, **FCR**, **FCR/bevacizumab**
**FCR**, **FCR**, OFAR, Lenalidomide/ofatumumab
Chlorambucil, CVP, FR
RCHOP, Rituximab, Rituximab/gemcitabine/oxaliplatin
Chlorambucil, Fludarabine, Alemtuzumab, PCR, Ofatumumab, **FCR**
FC, Hyper-CVAD, **FCR**
Rituximab/Steroids
FC, **FCR**, Lenalidomide/rituximab
Chlorambucil/rituximab, **FCR**, Lenalidomide
RCHOP, Nutlin
Fludarabine, Chlorambucil, **FCR**
Chlorambucil, RCVP, **FCR**
FR, Tositumomab/iodine I 131 tositumomab, Chlorambucil/rituximab, **FCR/bevacizumab**
**FCR**, **FCR**, Lenalidomide/rituximab
Rituximab/GM-CSF, **FCR**, Lenalidomide/ofatumumab
Idelalisib/Rituximab
**FCR/GM-CSF**
Ofatumumab
**FCR**, **FCR/bevacizumab**, Allo-SCT, Lenalidomide
**FCR**, Lenalidomide
RCVP
**FCR**, Rituximab, bendamustine, RCVP
Chlorambucil, RCHOP, Rituximab
Rituximab

FCR or FCR-based regimens are in bold.
FCR: Fludarabine, cyclophosphamide, rituximab; OFAR: Oxaliplatin, fludarabine, cytarabine, rituximab; CFAR: Alemtuzumab, fludarabine, cyclophosphamide, rituximab; RCHOP: Rituximab, cyclophosphamide, doxorubicin, vincristine, prednisone; CVP: Cyclophosphamide, vincristine, prednisone; FR: Fludarabine,

rituximab, PCR: Pentostatin, cyclophosphamide, rituximab, FC: Fludarabine, cyclophosphamide; RCVP: Rituximab, cyclophosphamide, vincristine, prednisone; Allo-SCT: Allogeneic stem cell transplant

Supplementary Table 2. Course 1 Hematologic Toxicities During Phase I

		Percent of Patients						
		Neutrophils		Platelets		Hemoglobin		Dose reduction after course 1
Bendamustine Dose Level	N	G3	G4	G3	G4	G3	G4	
20 mg/m^2	6	--	50	17	--	--	--	--
30 mg/m^2	3	--	33	67	--	33	--	33
40 mg/m^2	6	17	50	17	17	17	--	50
50 mg/m^2	6	--	50	17	33	33	--	33

G=Grade of toxicity

Supplementary Table 3. Summary of Representative Clinical Trials of CIT in Relapsed CLL

Regimen	Reference	Enrollment Time Frame	N	No. of prior Therapies Median (range)	Age median (range) yrs	Unmutated *IGHV* %	del17p %	del11q %	ORR/CR %	PFS/OS Months	Grade ≥3 Neutropenia %	Grade ≥3 Thrombocytopenia %	Grade ≥3 Infections %	T-MDS/AML %	Completed 6 courses %
FCR (MDACC)	Badoux, Blood 2011; Wierda, JCO 2005	1999-2008	284	2 (1-10)*	60	69	--	--	74/30	20.9/46.7	81	34	16	3	42
FCR arm (REACH Trial)	Robak, JCO 2010	2003-2007	276	--#	63 (35-83)	61	7	21	70/24	30.6/not reached	89	27	19	NR	68
FCR arm (LUCID Trial)	Awan, BJH 2014	--	311	1 (1-6)	61 (34-82)	50	8	27	72/15	23.9/not reached	71	15	10	1	60
BR (GCLLSG)	Fischer, JCO 2011	2006-2007	78	2^ (1-5)	66.5 (42-86)	67	19	22	59/9	15.2/33.9	23	28	13	NR	56
PCR (MSKCC)	Lamanna, JCO 2006	2001-2004	41	2 (1-7)	62 (30-80)	--	--	--	75/25	25/44	53	16	28	0	72
CFAR (MDACC)	Badoux, Blood 2011	2002-2006	80	3 (1-14)	59.5 (39-79)	89	31	31	65/23	10.6/16.7	92	54	46	NR	18
FBR (MDACC)	Current Trial	2010-2013	51	2 (1-6)	62 (46-82)	79	9	42	67/36	19/32	76	49	33	6	12

* 65% had no prior rituximab; # Prior Rituximab was not allowed, Prior FC was not allowed; ^ 91% had no prior rituximab; @ Percentage is based on number of treatment courses (and not number of patients); NR: Not reported

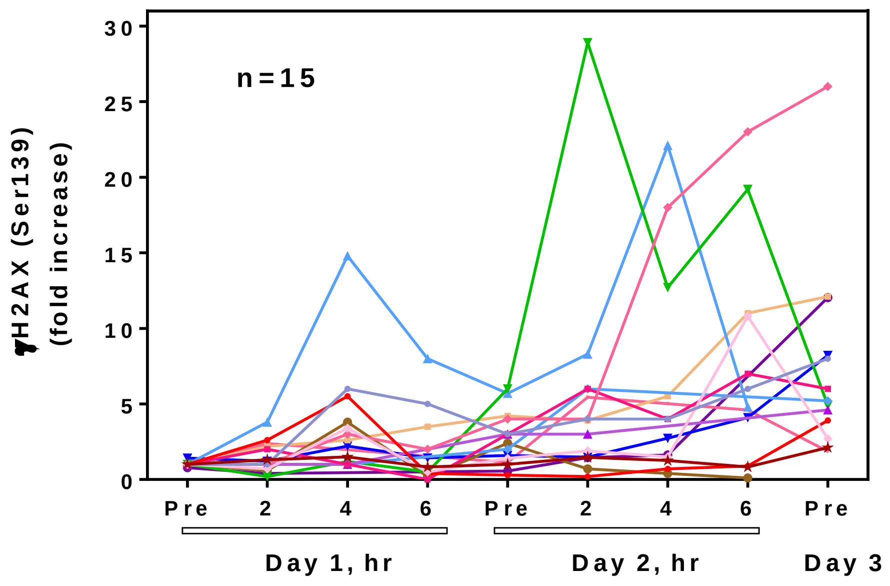

Supplemental Figure 1. DNA damage response measured as H_2AX phosphorylation: Blood samples were obtained during bendamustine and fludarabine + bendamustine therapy and CLL lymphocytes were isolated by ficoll gradient method and fixed with ice-cold ethanol (70%) and 4% PFA/PBS. The H_2AX phosphorylation (γH2AX) was determined by flow cytometry method (n=15) as described in materials and methods. Pretreatment value was taken as one and fold-increase in H2AX signal was plotted.
